# Early results for active infective endocarditis

**DOI:** 10.11604/pamj.2017.28.245.13518

**Published:** 2017-11-21

**Authors:** Mahdi Aithoussa, Noureddine Atmani, Reda Mounir, Younes Moutakiallah, Mehdi Bamous, Abdessamad Abdou, Fouad Nya, Anis Seghrouchni, Siham Bellouize, Mohamed Drissi, Mostafa Elouennass, Youssef Elbekkali, Abdelatif Boulahya

**Affiliations:** 1 Department of Cardiovascular Surgery, Mohammed V Teaching Military Hospital, Hay Riad, 10100 Rabat, Morocco; 2 Faculty of Medicine and Pharmacy of Rabat, Mohammed V University, Souissi, Madinat Al Irfane, 10100 Rabat, Moroc; 3 Intensive Care of Cardiovascular Surgery, Mohammed V Teaching Military Hospital, Hay Riad, 10100 Rabat, Morocco; 4 Department of Bacteriology, Mohammed V Teaching Military Hospital, Hay Riad, 10100 Rabat, Morocco

**Keywords:** Infective endocarditis, valvular surgery, active phase

## Abstract

**Introduction:**

Cardiac surgery is frequently needed during active phase of infective endocarditis (IE). The purpose of this study was to analyze the immediate and late results and determine the risk factors for death.

**Methods:**

We retrospectively reviewed 101 patients with IE operated in the active phase. The mean age was 40.5 ± 12.5 years. 16 patients (15.8%) were diagnosed with prosthetic valve endocarditis (PVE). 81 (80.9%) were in NYHA functional class III-IV. Blood cultures were positive in only 24 cases (23.9%).

**Results:**

in-hospital mortality rate was 17.9% (18 cases). Multivariate analysis indentified five determinant predictor factors: congestive heart failure (CHF), renal insufficiency, high Euroscore, prolonged cardiopulmonary bypass time (> 120 min) and long ICU stay. The median follow-up period was 4.2 (2-6.5) years. Overall survival rate for all patients who survived surgery was 97% at 5 years and 91% at 10 years.

**Conclusion:**

Despite high in-hospital mortality rate, when patients receive operation early in the active phase of their illness, late outcome may be good.

## Introduction

In developing countries like ours, Infective Endocarditis (IE) occurs most frequently in patients with rheumatic heart disease (RHD) [1,2] who always have hard an unhealthy oral hygiene and neglege prophylaxis of IE. Guidelines for prevention and management of IE recommend early heart surgery [3,4], because those patients are still initially managed in community hospital with no tools for diagnosis such as laboratory investigations, imaging techniques. They are frequently referred to specialized centers in an advanced stage with severe valvular damage and hemodynamic impairment [5]. Despite a better knowledge of predisposing factors [6,7] refinements in diagnostic techniques, availability of new antibiotics [8] and a progress in surgical therapy, IE is associated with substantial morbidity and mortality [9,10]. Some factors are described as determinant of morbidity and mortality. In this study we evaluate the impact of some preoperative and preoperative clinical parameters on immediate results in patients who underwent surgery.

## Methods

The study was designed as a retrospective observational single-center study, which had been approved by our local medical ethic committee.Data were collected from a computed registry database of our cardiac surgical patients.Inclusion criteria were; definite infective endocarditis, according to the modified Duke criteria [11].

The following data were collected

*Baseline demographics:* age, gender, BMI (body mass index), clinical symptoms, abnormalities of ECG (electrocardiogram), comorbidities (diabetes mellitus, treatment of steroid, renal failure), the portal of entry previous medical procedure (dental, gastrointestinal, genitourinary, or cardiac.

*IE complications:* severe valvular dysfunction, heart failure, severe sepsis, neurological complications, digestive embolism and arterial embolism. Laboratory findings, microbiological data, transthoracic echocardiography (TTE) and transoesophageal echocardiography (TEE) were performed to confirm the diagnosis of IE.Particular care was taken to research the presence and location of vegetations. The mobility and echolucency of vegetations were evaluated in semi quantitative method, according to sanfilippo et al [12]. The presence and extent of abscesse and others mechanical complications (aorto-cavity fistula) were also assessed. Valve regurgitation was assessed by semi quantitative method: absent, mild (2+) or moderate-severe (3+ or 4+). All patients were operated upon with extracorporeal circulation in mild systemic hypothermia (32°), myocardial protection with topical cooling and antegrade infusion of cold crystalloid cardioplegia, but since 2000, the cold blood cardioplegia was routinely used. When the Infection is limited to the cusp of the native valve, complete removal of the valve and implantation of a mechanical valve usually resolves the problem. The most important aspect in the surgical treatment of these patients is radical resection of all infected tissues. The presence of cardiac fistula or when extensive resection of necrotic tissue created the defect, needed reconstruction using a glutaraldehyde fixed bovine pericardium or fresh autologous pericardium. The conservative techniques were used only in situations in which the partial resection of the impaired leaflets. Direct sutures, closing with bovine pericardium flap. Aggressive debridement was needed in patients with prosthetic valve endocarditis (PVE). Intra operative variables of interest included the number and type of valves infected.

*Outcomes measures were:* elective or non-elective surgery, need for transfusion (> 2 units of packed red blood cells). Adverse events (Low output syndrome “LOS”, reexploration for bleeding, sepsis, cerebrovascular accident, renal insufficiency, and in-hospital mortality.

*Definitions:* endocarditis was labeled as “active” if the patient required operation prior to the completion of the antibiotic treatment (6-8 weeks) the term “healed“ endocarditis was used if the surgery was performed after the completion of antibiotic therapy.

**Ethics approval:** The study was conducted in accordance with the ethical rules of the Helsinki Declaration and Good Clinical Practice. The study protocol was approved by Mohammed V University medical-ethical committees.

## Results

In the period under investigation from January 1994 to December 2014, a total of 101patients with definite diagnosis of active infective endocarditis (AIE) [13] underwent valvular surgery under cardiopulmonary by pass (CPB). Clinical characteristics are listed in Table 1. In the study group, there were male 75 (74.3%) and female 26 (25.7%). The mean age was 40.5±12.5 years. Only patients with left sided IE were included. Probable source of infection was present in only 16 cases (15.8%). Of these patients requiring surgical intervention, the majority of patients 85 (84.2%) were diagnosed with native valve endocarditis (NVE) and 16 (15.8%) withPVE. RHD was the commonest underlying disease and was present in 79 (78.2%) patients. Bicuspid aortic valvewas present in 6 (5.9%). 81(80.8%) were in NYHA functional class III or IV at first admission in hospital. Among the laboratory parameters, anemia and raised C-reactive protein(CRP) level were present in the majority of patients 65 (64.8%). Blood cultures were positive in only 24 cases (23.9%). *Straphylococci* were the most frequent microorganisms being isolated in 10 cases (97 %), followed by streptococciin 8 cases. All patients were investigated by TTE while TEE was performed in 38(37.8%). Vegetations were observed in all patients, abscess was found in 13(12.8 %). 10(%) Patients had cuspal or leaflet tear causing valvular regurgitation. The site of endocarditis was aortic valve in 47 (46.5%) patients, mitral in 30 cases (29.6%) and both aortic and mitral site in 24 patients (23.7%).Among patients with PVE, 7 has dehiscence prosthetic valve. Table 2 showed operative and postoperative data.The overall complication rate was high 87 (86.2%). CHF was the most prevalent which was observed in 33 patients (32.7%) developed perivalvular abscess. Visceral infractions were identified in 7 (6.9%) patients (splenic in 3 cases, renal in 3 cases and hepaticin one case) 2 (1.98%) patients developed coronary embolism. Mycoticaneurysm was diagnosed in 2 (1.98%) patients. An uncontrolled sepsis occurred in 8 (7.9%) patients. No elective surgery was performed in 25 patients (24.8%). The major indication for surgery was the presence of refractory heart failure in 32 (31.7%). The mean CPB time and aortic cross clamp time were 117.8±58,3mn and 78.8±41.5mn respectively. The median mechanical ventilation duration was 11 (5-20.5) hours and the median ICU stay was 48 (44-72) hours. In hospital mortality was 17.9% (18 cases). The most common causes of death were: sever sepsis (6 cases), low output syndrome (4 cases), multiorgan failure (MOF) (4 cases), intracranial hemorrhage (3 cases) and sever arrhythmia 1 case. Hospital death occurred in18 cases including 13 patients with NVE (12.9%) and 5 patients with PVE (4.9%). Main significant factors associated with in-hospital mortality in univariate analysis are presented in Table 3 including: CHF, preoperative renal insufficiency, left bundle branch block, prolonged CPB and prolonged aortic cross clamp, comorbidities (increased Euroscore), prolonged ventilation support, and long ICU stay. But multivariate analysis including all significant variables in univariate analysis identified only five independent predictor factors (Table 4). They were CHF, renal insufficiency, prolonged CPB time, high Euroscore, and prolonged ICU stay. Among 83 survivors, 4 patients died, 37 were alive and 42 were lost. Median follow-up period was 4.2 (2-6.5) years.Overall survival rate for all patients who survived surgery was 97% at five years and 91% at 10 years.

**Table 1:** Demographic data

Variables	Mean ± SD / n(%)
**Age (years)**	40.5 ± 12.5
**Gender**	
Male n (%)	75 (74.3%)
Female n (%)	26 (25.7%)
**BMI**	23.44 ± 8.71
**Diabetes n(%)**	4 (3.96%)
**HTA n(%)**	8 (7.9%)
**Smoking**	18 (17.8%)
**NYHA**	
III- IV	81 (80.2%)
CTI	0.58±0.64
**Impaired renal function n(%)**	24(23.8%)
**Embolism**	
Brain	6 (5.94%)
Spleen	3 (2.97%)
Hepatic	1 (0.9%)
Renal	3 (2.97%)
Coronary	2 (1.98%)
Mycoticanevrysm	2 (1.98%)
**Blood culture positive n(%)**	20 (19.80%)
**Euroscore**	5.76±3.94
**Ejection fraction < 40%**	12(11.8%)
**SPAP (mmHg)**	53.53 ± 21.12
**Valve involved**	
Mitral	30 (29.7%)
Aortic	47 (46.7%)
Mitral + aortic	24 (23.7%)
**NVE (%)**	16 (15.8%)
**PVE (%)**	85 (8.49%)

**BMI**: body mass index; **CTI**: cardiothoracic index; **NVE**: native valve endocarditis; **PVE**: prosthetic valve endocarditis; **SPAP**: Systolic pulmonary arterial pressure

**Table 2:** Operative data

Variable	Mean ± SD / n(%)
No-elective surgery n(%)	25 (2.48%)
CPB time (min)	117.8± 58,3
Aortic cross clamp time (min)	78,89 ± 41.59
Mechanical ventilation time (hours)	11(5-20.5)
ICU stay (hours)	48(44-72)
Post-operative hospital stay (days)	14± 11.2
LOS: n(%)	23 (22.9%)
Post-operative renal insufficiency n(%)	25(24.8%)
Reexploration for bleeding n(%)	6(5.9%)
Sepsis n(%)	8(7.9%)
Digestive complications n(%)	5(4.9%)
MOF: n(%)	15(14.9%)
In-hospital mortality n(%)	18 (17.8%)

**CPB**: cardiopulmonary bypass; **ICU**: intensive care unit; **LOS**: Low output syndrome; **MOF**: Multiorgan failure

**Table 3:** Univariate analysis

Variable	OR	IC à 95%	P
RI	0.34	0.11 - 1.04	0.05
CHF	0.19	0.07 - 0.56	0.003
Left BBB	0.46	0.21 - 1	0.05
Euroscore	0.78	0.68 - 0.9	0.001
Age	0.97	0.93 - 1.02	0.3
Sexe	2.14	0.73 - 6,2	0.16
LV	0.61	0.14 - 2.5	0.5
CPB time	0.98	0.97 - 0.99	0.001
Mechanical VA	0.97	0.96 - 0.99	0.011
ICU stay	0.99	0.98 - 1	0.011
PVE	1.6	0.89 - 3.1	0.1

**BBB**: bundle branch block; **CHF**: congestive heart failure; **CPB**: cardiopulmonary bypass; **ICU**: intensive care unit; **LV**: left ventricle; **PVE**: prosthetic valve endocarditis; **RI**: renal insufficiency

**Table 4:** Multivariate analysis

Variable	OR	IC à 95%	P
CHF	0.57	0.06-5.02	0.018
Euroscore	1	0.77-1.4	0.008
CPB time	0.97	0.94-1	0.0001
ICU time	1	0.99-1.01	0.0001
RI	0.11	0.02-0.55	0.008

**CHF**: congestive heart failure; **CPB**: cardiopulmonary bypass; **ICU**: intensive care unit; **RI**: renal insufficiency

## Discussion

Active infective endocarditis (AIE) remains a devastating disease with high morbidity and mortality, which generally needs surgery and appropriate antimicrobial therapy. Surgery is potentially life saving [14] and is required in 25 to 50% of cases during acute infection and 20 to 40% during convalescence [15,16]. The profile of the disease differs among developed and developing countries. Even though the diagnostic tools and medical center are comparable to those used in western countries, our study showed some important differences between results and western reports. Our study investigated a selected group of patients suffering from AIE who underwent valve surgery.In this study, patients are mostly young (mean age 40.5 ± 12.5 years) with 49 (48.5%) below 40 years of age. This findings are similar to that reported by Fragomeni [5], but higher when compared to recent Indian studies [17,18] 27±12.7years and 23.5 years respectively. This age is similar to that reported in recent clinical epidemiologic study conducted in Laos' speoplein democratic republic in Vietnam (25 years) [19]. The low age 32.4 years was also observed in Tunisian study published by Letaief [20]. This is in contrast to the west where majority of patients present beyond the five decade.Recent studies from developed countries found the mean age higher than 60 years [21-23]. Chronic rheumatic disease was the most frequent underlying heart disease in our study as well as in other studies from this part of world. However, in some middle-income country, the incidence of RHD has declined during the last decade [18,19,20,24,25]. In developed countries there is a decrease in RHD and increase in degenerative heart disease.According to the NYHA functional class classification, most of patients 81(80.2%) were in class III-IV on the admission to the hospital. Similar data were observed in other investigations [5,17,24,26]. It is difficult to determine the real prevalence of IE in the general population because this disease is continuously changing.In recent retrospective study conducted in 9 French ICU during an 11- year period, Duval et al [27] found the annual incidence of IE at the beginning of the 21^st^ century is around 33 case per million habitants. The most characteristic finding in our study was the high frequency of negative blood culture 77(76.2%). Our results were in accordance with previous reports from the developing world [5,18,19,20,28]. In contrast, in series from western countries, blood cultures were found negative in 5-15% of IE [23]. Many factors are incriminated in the high frequency of IE negative blood culture the discriminate use of antibiotics in the initial phase of symptoms is the major cause of lack of growth of microorganisms in culture. Some patients may be treated outside partially or inadequately. They probably had infection with less virulent and susceptible organisms leading to culture negatively.As observed in most developing countries, antibiotics are available and self-medication in common and may have contributed to the low frequency of positive blood cultures [29]. Lack of availability of advanced technologies may identify rare and fastidious microorganisms. In this study, in patients with positive blood culture or culture of explanted valve or prosthesis, *streptococci and staphylococci* were the most frequent causative agents. These findings are consistent with other reports [19,20,23,26,30]. As described in other series [31], the high prevalence of *streptococcal pathogen agent* in our study reflects poor oral and dental health. The majority of our patients were diagnosed with native valve endocarditis 85 (84.2%). Our findings are similar to previous reports [19,20,32-34].

The indication for the surgical treatment of IE reported in the literature are similar to those in our study [23,35]. Despite advances in diagnosis and medical management, the in-hospital surgical mortality rate remains high, since ranging from 15 to 22% [19,26,27,30,35]. Agca and al [24] found high in hospital mortality rate (36%). In our serie, overall surgical mortality rate was 17.8%. Our findings are consistent with various publishing reports. Operative mortality variesvariedly in the literature because of the differences in the study population (developed/developing countries) and the heterogeneity of this disease. Assessment of the impact of surgery on outcome is difficult. In general terms, prognosis is better if surgery is undertaken early, before cardiac tissue destruction and deterioration in the overall condition of the patient.Timing of surgery remains controversial in this population, as there are no randomized control trials that document short and long term mortality.Early surgical intervention has been reported as a protective factor for mortality in recent studies, which is concordant with our result [36]. Clinical factors including, CHF, periannular complications and *staphylococcus aureus* infections have been reported in prospective trials to benefit from earlier surgical intervention [37]. The impact of surgery on IE prognosis was the subject numerous studies. Despite some conflicting results, surgical therapy appears most often associated with an improved early and late survival in the overall population [19,38,39] several demographic and clinical factors have been associated with adverse outcome in patients with AIE. In present study, independent predictors of mortality in multivariate analysis were: CHF, preoperative renal insufficiency, high Euroscore, prolonged CPB (> 120 mn), prolonged mechanical ventilation and long ICU stay. The operative mortality of surgery in active IE ranges from 5% to 10% in patients without CHF and from 15% to 35% in patients with CHF [40]. During the past few years, several published reports have identified a number of prognostic factors related to higher mortality, such as advance age [16,26,37,41]. Prosthetic valve endocarditis and *staphylococcus* agent were also predictors of high mortality [16,41]. Patients with evidence of left ventricular dysfunction had a higher risk of death [26,32,42]. In our study, we failed to find the relationship between impaired left ventricular function, PVE, and staphylococcus and early worse outcomes. Neurologic dysfunction complicates the course of 10-40% of left sided IE and was associated with increased mortality [9,43,44]. In our serie, six (6.95%) patients developed cerebral embolic complication but one patient experienced postoperative neurological deterioration resulting from the exacerbation of intra cerebral hemorrhage. In recent published study, Fabio chirillo [42] found that surgical mortality was significantly higher among diabetic patients (34%) that in non-diabetic (20%). Agca and al [24] also found diabetes as a predictor factor of high operative death.The late survival outcome after surgical treatment for AIE is known to be good with reported 10 years rate ranging from 52% to 71.3% [45,46].

**Study limitations:** The present study has several limitations. First, it was a retrospective study performed only at a single institution. Second, the sample size was relatively small and was need larger sample with more patients that may enable us to assess predictors of mortality. Third, our serie include both NVE and PVE, this may be overall mortality biased. Fourth, it is difficult to find a relationship between high mortality and infecting agent because of high frequency of negative blood culture. Other performing tool could resolve this problem (like PCR).

**Funding:** We declared that the present research not received subvention or any funding from elsewhere source, private or public.

## Conclusion

Despitea high in-hospital mortality rate, early surgical strategy for active infective endocarditis seems to be acceptableto prevent preoperative adverse and the late outcome may be good

### What is known about this topic

- Active infective endocarditis (AIE) remains a devastating disease with high morbidity and mortality;
- Early surgical intervention has been reported as a protective factor for in-hospital mortality;
- Most frequent prognostic factors related to higher mortality were: advanced age, prosthetic valve endocarditis, staphylococcus agent and prosthetic valve endocarditis.

### What this study adds

- Our study showed some important differences between results and western reports: our patients were younger and chronic rheumatic disease was the most frequent underlying heart disease;
- The most characteristic finding in our study was the high frequency of negative blood culture;
- In-hospital surgical mortality rate remains high, but the late outcome seems to be good.

## Competing interests

The authors declare no competing interests.
